# JAK2 inhibitor persistence in MPN: uncovering a central role of ERK activation

**DOI:** 10.1038/s41408-022-00609-5

**Published:** 2022-01-26

**Authors:** Garima Pandey, Andrew T. Kuykendall, Gary W. Reuther

**Affiliations:** 1 Department of Molecular Oncology, Moffitt Cancer Center, Tampa, FL USA; 2 Department of Malignant Hematology, Moffitt Cancer Center, Tampa, FL USA

**Keywords:** Preclinical research, Targeted therapies, Cancer therapeutic resistance, Myeloproliferative disease, Oncogenes

## Abstract

The Philadelphia chromosome negative myeloproliferative neoplasms, including polycythemia vera, essential thrombocytosis, and myelofibrosis, are driven by hyper activation of the JAK2 tyrosine kinase, the result of mutations in three MPN driving genes: *JAK2*, *MPL*, and *CALR*. While the anti-inflammatory effects of JAK2 inhibitors can provide improved quality of life for many MPN patients, the upfront and persistent survival of disease-driving cells in MPN patients undergoing JAK2 inhibitor therapy thwarts potential for remission. Early studies indicated JAK2 inhibitor therapy induces heterodimeric complex formation of JAK2 with other JAK family members leading to sustained JAK2-dependent signaling. Recent work has described novel cell intrinsic details as well as cell extrinsic mechanisms that may contribute to why JAK2 inhibition may be ineffective at targeting MPN driving cells. Diverse experimental strategies aimed at uncovering mechanistic details that contribute to JAK2 inhibitor persistence have each highlighted the role of MEK/ERK activation. These approaches include, among others, phosphoproteomic analyses of JAK2 signaling as well as detailed assessment of JAK2 inhibition in mouse models of MPN. In this focused review, we highlight these and other studies that collectively suggest targeting MEK/ERK in combination with JAK2 inhibition has the potential to improve the efficacy of JAK2 inhibitors in MPN patients. As MPN patients patiently wait for improved therapies, such studies should further strengthen optimism that pre-clinical research is continuing to uncover mechanistic insights regarding the ineffectiveness of JAK2 inhibitors, which may lead to development of improved therapeutic strategies.

## Introduction

Philadelphia chromosome-negative myeloproliferative neoplasms (MPNs) are a family of neoplastic myeloid diseases that initiate in the bone marrow from aberrant regulation of hematopoietic stem cells [1]. Mutations that activate signaling via the JAK2 tyrosine kinase drive MPN formation - resulting in clonal hematopoiesis and trilineage myeloproliferation leading to leukocytosis, thrombocytosis, and erythrocytosis [2–4]. MPNs include polycythemia vera (PV), essential thrombocytopenia (ET) and primary myelofibrosis (PMF). PV and ET each have a prevalence of ~160,000 in the U.S., while PMF has a lower prevalence (~16,000) [5], in part due to a much worse prognosis. Diagnostic hallmarks of MPNs include megakaryocyte hyperplasia and elevated platelets in ET patients, panmyelosis and elevated hematocrit in PV patients, and megakaryocyte atypia and marrow fibrosis in PMF. Despite these distinctions, these diseases represent a continuum of overlapping hematological phenotypes with many patients experiencing a transition in their MPN during the course of their disease; evolving from ET to PV or from either ET or PV to myelofibrosis. Furthermore, the newly-adopted diagnostic entity of pre-fibrotic myelofibrosis, which demonstrates histomorphology consistent with PMF despite lacking moderate to severe marrow fibrosis, further exemplifies the phenotypic continuum of MPNs [6–8].

Historically MPNs were diagnosed based on morphologic assessment of the bone marrow and correlating peripheral blood values. More recently, the presence of recurrent genetic driver mutations has become a key criterion [4]. JAK2 activating mutations in MPN were first identified in 2005 with the discovery of a point mutation leading to a JAK2-V617F mutant protein that exhibits elevated activity, leading to deregulated signaling and hypersensitivity to cytokines [9–12]. JAK2-V617F is present in 95% of PV patients and 50–60% of ET and myelofibrosis patients. Assessment of MPN patients lacking this JAK2 mutation led to the identification of activating exon 12 *JAK2* mutations in PV [13], as well as mutations in the thrombopoietin receptor (MPL) [14] and in the endoplasmic reticulum chaperone protein calreticulin (CALR) in ET and PMF [15]. *JAK2*, *MPL*, and *CALR* mutations each induce JAK2 activation and signaling [2] and induce MPN phenotypes in mice [16], demonstrating such mutations are drivers of MPN. Interestingly, JAK2 functions downstream of MPL, and MPN-driving mutant CALR proteins interact with MPL and induces activation of JAK2 signaling [17]. Thus, from a protein-centric perspective MPL is a key player in driving MPN. This is supported by the observation that megakaryocytes, which are regulated by MPL signaling, are critical driving cells of MPN [18–23]. While true, MPL and CALR mutations predominantly drive ET and myelofibrosis likely due to selective expression of MPL in hematopoietic stem cells (HSCs), multipotent progenitors, common myeloid progenitors (CMPs), megakaryocyte erythroid progenitor cells (MEPs), and megakaryocyte precursor cells. While PV-driving JAK2 mutations may also alter regulation of HSCs due to MPL expression, these mutations can deregulate signaling from cytokine receptors such as GM-CSFR, GCSFR, IL3, and EPOR, that are expressed in CMPs, granulocyte macrophage progenitor cells, MEPs, and erythroid progenitor cells [24]. Thus, through overlapping and unique cellular effects, MPN driving mutations lead to the continuum of MPN phenotypes in part based on the context of cell type and cytokine receptor expression in hematopoietic stem and progenitor cells.

Based on a plethora of pre-clinical studies, and close analogy to imatinib in CML (i.e., a driver kinase inhibitor therapy in a chronic myeloid neoplasm), MPN patients were seemingly primed to be successfully treated by JAK2 inhibitors. However, comparative success has not been attained, as cells containing the JAK2-activating mutation persist in patients undergoing JAK2 inhibitor therapy, and unlike imatinib in CML, JAK2 inhibitors do not induce remission. Understanding mechanisms by which MPN-driving cells persist in the face of chronic JAK2 inhibition could lead to alternative strategies for targeted therapy-based approaches for MPN patients. Herein we discuss JAK2 inhibitor persistence and link numerous studies that use disparate approaches yet converge on highlighting a potential critical role for the MEK/ERK pathway in the persistent survival of MPN-driving cells during chronic JAK2 inhibitor therapy.

### Anti-JAK2 targeted therapies for MPN

Two JAK2 inhibitors, ruxolitinib and fedratinib, have been FDA approved for patients with intermediate and high-risk myelofibrosis, while ruxolitinib is also approved for hydroxyurea intolerant PV patients. Ruxolitinib has been in clinical use for a decade and hence a lot more is known about its long-term clinical responses than the more recently approved fedratinib. Ruxolitinib and fedratinib have little initial effect on reducing mutant JAK2 allele burden [25, 26] suggesting JAK2 inhibitors may be limited in their capacity to reverse the course of disease and induce remission in patients. JAK2 inhibitor approval in MPNs was based on anti-inflammatory effects that reduce spleen size and improve disease-related symptoms. Despite little impact on mutant allele burden in the short-term, long-term studies have indicated that ruxolitinib has the potential to decrease the *JAK2* mutant allele burden, improve the extent of bone marrow fibrosis, and increase the survival of those patients who can remain on ruxolitinib for years [27–33]. This suggests that anti-JAK2 therapies have the *potential* to antagonize the dominancy of the malignant clone and revert the natural course of disease. This is important considering that additional JAK2 inhibitors, which have demonstrated unique properties that should allow the expansion of JAK2 inhibitor use to clinically distinct patient populations (e.g., pacritinib in patients with low platelets), are seemingly on the horizon for patients [34].

### JAK2 inhibitor resistance/persistence in MPN

Due to their favorable impact on spleen volume and disease-related symptoms, as well as potential survival benefits, JAK2 inhibitors such as ruxolitinib will likely remain a mainstay in the treatment of patients with MPN [27–33]. Nevertheless, JAK2 inhibition is suboptimal for most patients for several reasons. Some patients with myelofibrosis do not have splenomegaly or significant symptom burden, instead, featuring problematic cytopenias that are likely to be exacerbated by JAK2 inhibitors. In those that are appropriate for JAK2 inhibition, approximately half will discontinue JAK2 inhibitor by 3 years [35]. Reasons for discontinuation at 3 years include intolerance, suboptimal response, loss of response, refractory disease, and progressive disease. Thus, short- and long-term benefits of treatment, including potential survival benefits, are not possible for many patients. Finally, while ruxolitinib can provide a survival benefit, there is no clear evidence that ruxolitinib therapy in myelofibrosis patients significantly affects the rate of transformation to AML [29, 36–38]. Collectively, the clinical experience of JAK2 inhibitors, mostly with ruxolitinib, indicates that targeting JAK2 alone does not readily induce a reduction of MPN-driving allele burden suggesting this therapeutic treatment has little effect on disease-driving stem cells and is unlikely to induce remission.

While JAK2 inhibitor resistance in patients is tangible, it is rather ill-defined, and it is poorly understood at the molecular level. JAK2 mutations that confer resistance to JAK2 inhibitors have been identified in genetic screens in cell lines [39], but such mutations are essentially never found in patients and thus play little role in the response of patients to JAK2 inhibitors or clinical management [40, 41]. The clinical response of JAK2 inhibitors is based on the anti-inflammatory activity of these drugs which provides patient quality-of-life benefits. Thus, clinical resistance is the absence of an amelioration of the effects of the inflammatory state associated with MPNs [42, 43], including splenomegaly and constitutional symptoms. Importantly, clinical resistance may be JAK2 inhibitor specific, as some patients who fail ruxolitinib can achieve clinical benefit with fedratinib treatment [44, 45]. This further supports the development of additional JAK2 inhibitors for MPN patients, several of which are advancing toward approval [34].

JAK2 inhibitor persistence in MPN was originally described by Koppikar et al. [41] who demonstrated that MPN model cells could be made to grow in high concentrations of ruxolitinib (and other type 1 JAK2 kinase inhibitors) and that this drug resistant state was reversible—cells cultured after removing the drug regain their sensitivity to JAK2 inhibitors. This demonstrated non-genetic mechanisms contributed to cell survival and proliferation in ruxolitinib. Both stabilization of JAK2 protein and an increase in JAK2 transcription was suggested to play a role in the persistent growth of cells cultured with JAK2 inhibitors. It was also demonstrated that ruxolitinib persistence was associated with an induction of heterodimeric complexes of JAK2 with other JAK family members, notably JAK1 and TYK2, and that this heterodimerization could reactivate JAK2 signaling [41]. The fact that ruxolitinib is also a potent JAK1 inhibitor suggests that there are more details to be understood about the role of such heterodimerization. More recently, it was reported that the stabilization of JAK2 protein observed during ruxolitinib treatment is due to the insensitivity of ruxolitinib-bound JAK2 to dephosphorylation by phosphatases and subsequent ubiquitination and degradation [46]. Thus, ruxolitinib itself may be inducing structural changes in JAK2 and/or JAK2 protein complexes that alter the regulation of JAK2 protein expression and activity, contributing to JAK2 inhibitor persistence. Inhibition of JAK2 protein levels by HSP90 inhibition is one approach to counteract deregulated JAK2 protein expression during JAK2 inhibitor persistence [41, 47, 48]. Genetic removal of JAK2 in MPN mouse models has indicated JAK2 remains requisite for JAK2 inhibitor persistence [48], suggesting JAK2 protein is required to maintain a drug persistent state. While JAK2 bound to ruxolitinib should not be active, JAK2-dependent signaling proceeds during JAK2 inhibitor persistence, which is also evident in therapeutic mouse models of MPN—following an initial response to JAK2 inhibitor therapy, MPN phenotypes redevelop during JAK2 inhibitor monotherapy [49, 50].

The evidence is clear—MPN cells can persistently survive and proliferate during JAK2 inhibitor monotherapy—in MPN patients, cell lines, as well as in MPN mouse models. Combination targeted therapies as an upfront approach where the goal would be to prevent the development of JAK2 inhibitor persistence, or an add-on therapy where critical pathways that maintain persistence can be antagonized, may enhance the efficacy of JAK2 inhibitor therapy [51, 52]. Notably, this is exemplified by studies of ruxolitinib in combination with bromodomain inhibition or inhibition of the anti-apoptotic Bcl2 family members that are showing promising results in clinical studies in myelofibrosis patients [53–55]. Reviews of ongoing combination therapy studies for MPN have recently been published and will not be addressed here [51, 52].

A variety of experimental approaches have been used to identify potential mechanisms and therapeutic vulnerabilities of JAK2 inhibitor persistence in MPNs. In addition to candidate approaches such as targeting known downstream components of JAK2 signaling and epigenetic regulators (e.g., AKT, mTOR, PIM, PRMT5, LSD1, among others) [49, 51, 52, 56–63], less candidate-centric approaches, including phosphoproteomics [64], interrogation of JAK2 persistence in MPN mouse models [65] as well as gain of function genetic screens [66] have been used to identify potential mechanisms of JAK2 inhibitor persistence. The advantage of such approaches is that they have the potential to uncover unexpected mechanistic aspects and/or highlight signaling pathways that could contain targets for combination therapies to antagonize JAK2 inhibitor persistence. Interestingly, each of these approaches have led to similar findings—that MEK/ERK inhibition may thwart JAK2 inhibitor persistence and improve patient responses to JAK2 inhibitor therapy.

### Phosphoproteomic insight into JAK2-V617F signaling and response to JAK2 inhibition

**Fig. 1:**
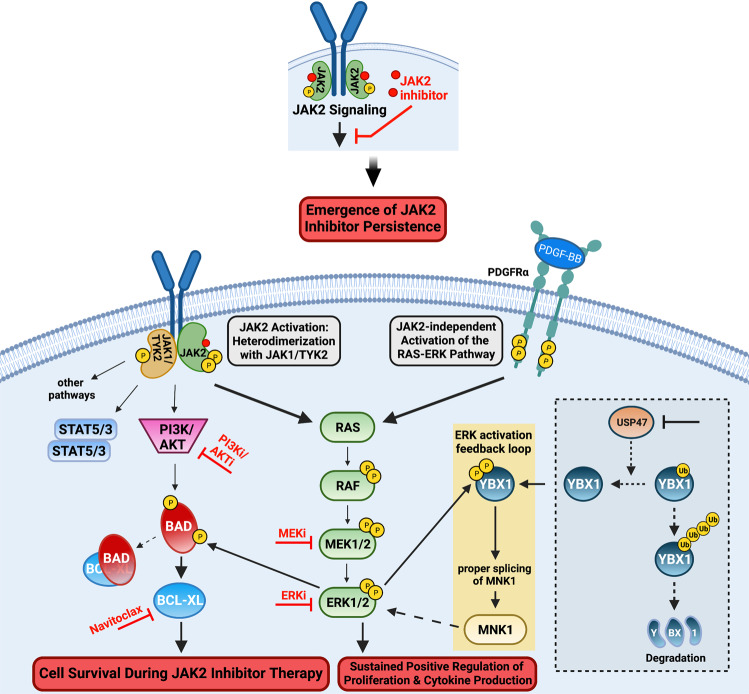
Central Role of MEK/ERK activation in JAK2 inhibitor persistence in pre-clinical models of MPN. Multiple approaches have been used to uncover mechanisms of JAK2 inhibitor persistence in models of MPN, including JAK heterodimerization to reactivate JAK2 signaling [41], upregulation of PDGFR signaling as a JAK2-independent mechanism to activate ERK [65], and regulation of YBX1 function required to maintain ERK activation via splicing of MNK1 [64]. BAD inactivating phosphorylation by AKT as well as ERK prevents apoptosis in the presence of JAK2 inhibitors. MEK and ERK inhibition as well as BCX-XL inhibition (navitoclax) may antagonize ERK-dependent effects on cell survival to improve the efficacy of JAK2 inhibitors. In analogy (shown by dashed box) to studies in imatinib resistance in CML, USP47 may control YBX1 protein levels [79], potentially providing a target to antagonize the role of YBX1 in JAK2 inhibitor persistence.

Recently, Jayavelu et al. [64] reported the first detailed global phosphoproteomic analysis of the JAK2 signaling landscape associated with JAK2-V617F and wildtype JAK2. Among the most enriched cellular processes associated specifically with JAK2-V617F signaling were those involved in mRNA splicing and processing. A small, focused RNA interference screen designed to target the top proteins identified in these pathways was performed to assess their potential roles in cell survival in response to ruxolitinib. From this, the loss of the protein YBX1 was identified as sensitizing cells to growth inhibition and induction of apoptosis by ruxolitinib without affecting cell growth in the absence of the JAK2 inhibitor—defining a potentially novel genetic-drug synthetic lethality. The genetic absence of *YBX1* in an MPN mouse model driven by JAK2-V617F prevented disease formation and importantly, also allowed for ruxolitinib treatment to lead to molecular remission, which was not seen when YBX1 was present. In addition, knockdown of YBX1 in a human JAK2-V617F model cell line sensitized these cells to JAK2 inhibitor treatment in vivo. These data suggest YBX1 plays a role in JAK2-V617F-driven disease and plays a role in the persistent survival of cells challenged with JAK2 inhibition (Fig. 1).

YBX1 is a nucleic acid-binding protein that has described roles in transcription as well as mRNA splicing and processing [67–72], and is involved in regulating the expression of cancer-associated drug resistance genes [73]. Notably, YBX1 was recently shown to enhance translation of cMYC in AML, contributing to a competitive proliferative advantage of malignant cells [74], and has been identified as a regulator of gene expression induced by MAPK [75]. It was shown that YBX1 was phosphorylated in a JAK2-V617F and MEK/ERK-dependent manner on serine residues 30 and 34, suggesting altered regulation of YBX-1 function in cells expressing JAK2-V617F [64]. In fact, the nuclear localization of YBX1 was shown to be enhanced by phosphorylation on these serine residues, and that YBX1 nuclear localization was retained in the presence of JAK2 inhibition but was antagonized by MEK inhibition. This suggests the nuclear function of YBX1 is more sensitive to MEK inhibition than JAK2 inhibition suggesting JAK2 inhibition alone may not antagonize the function of YBX1, potentially contributing to JAK2 inhibitor persistence.

Given the role of YBX1 in RNA splicing, alterations in mRNA splicing in JAK2-V617F cells were investigated. This led to the identification of a significant increase in intron retention, including in mRNAs that encode proteins involved in RNA splicing, nonsense-mediated decay, apoptosis, as well as MEK/ERK signaling [64]. One of these is MAPK-interacting kinase 1 (MNK1) whose expression was diminished in JAK2-V617F cells that were depleted of YBX1. MNK1 expression in JAK2-V617F expressing cells was shown to be dependent on YBX1 function and required for full ERK signaling (Fig. 1). MNK1 inhibition enhanced JAK2 inhibitor-induced apoptosis, and MEK inhibition synergistically induced apoptosis of primary CD34 + JAK2-V617F expressing cells. In PDX mouse models using primary human JAK2-V617F-expressing bone marrow of MPN patients, the combination of ruxolitinib and the MEK inhibitor trametinib significantly inhibited the growth of transplanted cells and induced molecular remission compared to mice treated with ruxolitinib alone. These experiments highlight the potential MEK inhibitors may have in combination with JAK2 inhibitors in MPN. Other interesting observations from this study [64] include interaction of JAK2 and MAPK1 (ERK2) and the loss of ERK phosphorylation following MNK1 knockdown. As MNK1 is known to be *downstream* of and a substrate for ERK activity, this observation was unexpected [76–78].

The study by Jayavelu et al. [64] concludes that YBX1 function, via proper splicing and subsequent expression of MNK1, is required to maintain ERK signaling by JAK2-V617F and to maintain ERK signaling in the presence of JAK2 inhibition, and impeding YBX1 expression (or its functional phosphorylation at ERK sites) deregulates MCL1 and BIM expression to favor apoptosis. Thus, the authors present a novel cell intrinsic mechanism by which JAK2-V617F signaling controls ERK activity via maintaining YBX1 in the nucleus to regulate, at least, the splicing of mRNAs of key signaling proteins (e.g., MNK1). This study indicates that therapeutically targeting MEK/ERK or MNK1 could antagonize ruxolitinib persistence and enhance the efficacy of JAK2 inhibitor therapy (Fig. 1).

In a recent study ubiquitin-specific peptidase USP47 was identified as a target to overcome tyrosine kinase inhibitor resistance in CML [79]. YBX1 was identified as a binding partner and substrate of USP47, whereby inhibition of USP47 destabilized YBX1 protein (via enhanced YBX1 ubiquitination). The loss of YBX1 was suggested to overcome tyrosine kinase inhibitor resistance, although this study indicates that YBX1 functions to mediate DNA damage repair and thus the loss of YBX1 via USP47 inhibition leads CML cells to cell cycle arrest and apoptosis. Thus, while there is no YBX1 inhibitor available, inhibitors of deubiquitinating enzymes such as USPs have been developed and as shown by Lei et al. [79] may be a strategy to target the function of YBX1 (Fig. 1). The expression of YBX1 was determined to be elevated in bone marrow from MPN patients (PV, ET, and myelofibrosis) compared to healthy bone marrow cells [64], suggesting cells from some MPN patients may be more sensitive to a strategy to target YBX1 function. Interestingly, using single cell RNA-seq Psaila et al. [80] identified YBX1 expression among several genes identified as potential regulators of differentiation of hematopoietic stem cells toward the megakaryocyte lineage, as compared to erythroid differentiation, in myelofibrosis patients and not healthy individuals. Given the disease-driving role of megakaryocytes in MPNs [18–23], these studies collectively suggest targeting YBX1 function may selectively antagonize megakaryocyte over erythroid development in MPN patients, and importantly, potentially spare healthy megakaryocyte development.

### Murine models of human MPN identify JAK2-independent signaling that mediates JAK2 inhibitor persistence

Utilizing in vivo mouse models of MPN, Stivala et al. [65] describe how cell extrinsic mechanisms could provide cell survival signals to MPN-driving cells in the presence of JAK2 inhibitors. This study demonstrated that while ruxolitinib-mediated inhibition of ERK activation is observed in cell lines as well as in MPN patient and mouse model cells treated with ruxolitinib ex vivo, ERK remains activated in JAK2-V617F and MPL-W515L MPN model mice therapeutically treated with ruxolitinib. Phospho-receptor tyrosine kinase arrays and multiplexed RNA expression analyses were used with bone marrow cells and splenocytes of JAK2-V617F and MPL-W515L mice to identify receptor and extracellular ligands that could potentially drive compensatory ERK activation during JAK2 inhibitor therapy. This led to the identification of PDGFRα activation, via its ligand PDGF-BB, as a potential mediator of extracellular-initiated signaling that could lead to JAK2-independent ERK activation during ruxolitinib therapy in vivo. Interestingly, both PDGF ligands and PDGFRα were induced by ruxolitinib treatment in megakaryocyte-erythroid progenitor cells suggesting that ruxolitinib therapy can *induce* activation of this RTK pathway in a relevant disease-driving cell type. PDGFRα expression was also induced in other subtypes of bone marrow cells including LSK stem cells and CMPs, among others. Thus, cellular responses to ruxolitinib may include rapid induction of RTK-mediated signals, such as PDGFR signaling, that antagonize the effect of ruxolitinib on JAK2 signaling, potentially reducing the upfront efficacy of ruxolitinib therapy, and allowing for continued persistent survival during JAK2 inhibitor therapy (Fig. 1). However, it should be noted that the involvement of PDGFR signaling in ruxolitinib persistence in primary MPN patient cells remains to be described.

Combined JAK2 and MEK inhibition in JAK2-V617F mice was shown to suppress activation of both ERK and STAT5, unlike JAK2 inhibitor therapy alone [65]. The combination treatment also normalized manifestation of disease including greater effects on antagonizing splenomegaly and hematocrit compared to monotherapies in this PV model. Multipotent myeloid progenitor, erythroid progenitor, and MEP cell frequencies, which are elevated in JAK2-V617F mice, were decreased to a greater extent upon combined JAK2 and MEK treatment than with each of the inhibitors alone. Importantly, bone marrow fibrosis was effectively resolved, and normal splenic cellular architecture was restored by the combination treatment but not by each treatment alone. Compared to monotherapies, the combination of MEK and JAK2 inhibition had a more pronounced inhibitory effect on the expression of inflammatory cytokines as well as ERK target genes, further demonstrating that JAK2 inhibition alone by ruxolitinib does not effectively inhibit ERK signaling in vivo. Qualitatively similar relative therapeutic efficacy of the combination therapy was observed in a MPN mouse model driven by MPL-W515L. In summary, this study suggests JAK2 independent activation of ERK can be induced by JAK2 inhibitors and may function as a compensatory mechanism employed by MPN cells to survive in the presence of JAK2 inhibition, thereby reducing effectiveness of anti-JAK2 targeted therapies. A follow up study [81] confirmed these results using genetic and pharmacologic inhibition of ERK activity, further exemplifying the notion that MEK/ERK signaling can antagonize the effect of JAK2 inhibitors, providing strong support for combining MEK or ERK inhibitors with JAK2 inhibitors in MPN (Fig. 1).

### Gain of function screens identify signaling pathways that antagonize the effects of JAK2 inhibition

In a more direct approach to determine what signaling pathways could antagonize JAK2 inhibition and induce JAK2 inhibitor resistance, Winter et al. [66] performed a screen using gain of function mutants of signaling proteins. Activating mutants of RAS, MEK, and AKT were identified and subsequently determined to antagonize JAK2 inhibition in multiple MPN model cell lines. The growth of JAK2 inhibitor persistent cell lines was shown to be sensitive to AKT inhibition and the combination of AKT and MEK inhibition further reduced the GI_50_ of JAK2 inhibitors. This study demonstrated that inactivating phosphorylation of the pro-apoptotic BAD protein was the key determinant of cell survival in response to JAK2 inhibition. JAK2 signaling can lead to phosphorylation and inactivation of the pro-apoptotic protein BAD via ERK, as well as AKT and PIM activity, which have also been investigated as therapeutic targets in MPN [48, 49, 51, 52, 58, 61]. In response to JAK2 inhibitor treatment, compensatory signaling that activates ERK and/or AKT could lead to inactivation of BAD and a subsequent loss of apoptosis in response to JAK2 inhibition. These data suggest that alternative signals induced in response to JAK2 inhibition (for e.g., RTK activation, etc.) could create a cellular state that is resistant to the effects of JAK2 inhibitors via compensatory inactivation of BAD activity, which is required for cell death induced by JAK2 inhibition. This study [66] suggested concomitant treatment with inhibitors of ERK activation (or AKT activity) could provide more durable responses in patients undergoing JAK2 inhibitor therapy. These could include MEK, in congruence with Stivala et al. [65], or ERK inhibitors as shown by Brkic et al. [81], as well as inhibitors of other signaling proteins that induce RAS activity. Of note, ongoing studies in our lab have shown potential for inhibition of SHP2, which functions upstream of RAS, to enhance the effectiveness of JAK2 inhibition in pre-clinical MPN models [82]. The convergence of JAK2 and compensatory RAS signaling during JAK2 inhibitor resistance on the inactivation of BAD function, which would leave the anti-apoptotic activity of BCL-2 proteins intact, nicely complements the promising early results of ongoing clinical studies assessing BCL-2 family inhibition with navitoclax in combination with ruxolitinib in myelofibrosis [54]. While navitoclax inhibits multiple BCL-2 family members, Waibel et al. [83] demonstrated that the BCL-2 family member most important downstream of JAK2 signaling and in JAK2 inhibitor persistence is BCL-XL, a finding confirmed by Winter et al. [66]. These preclinical studies and ongoing clinical trial data suggest impeding the anti-apoptotic nature of BCL-XL, and perhaps other BCL-2 family members, may improve JAK2 inhibitor therapy for myelofibrosis, and perhaps other MPN, patients (Fig. 1).

### Other evidence suggesting a role for RAS/MEK activation during JAK2 inhibitor persistence

*Genetic assessment of progressive disease in myelofibrosis patients: identification of the acquisition of RAS/RTK pathway mutations*: Mylonas et al. [40] utilized whole exome sequencing and single cell genotyping to interrogate the clonal evolution of myelofibrosis during ruxolitinib therapy. Interestingly, 2 of 15 patients in this study attained molecular remission during therapy, an exciting finding given the dearth of evidence for ruxolitinib to induce remission. Additionally, in one patient this study identified a JAK2-R867Q mutation, a mutation that is known to induce JAK2 inhibitor resistance [84]—a rare example of the acquisition of a second site *JAK2* mutation that could instill non-responsiveness to JAK2 inhibitors. One-third of the patient samples showed enrichment of mutations in RAS/RTK pathway genes that were obtained over time. Three patients studied developed progressive disease (e.g., leukemic transformation) and this was associated with the acquisition of *NRAS* or *KRAS* mutations over time in each patient. Whether or not the acquisition of RAS/RTK pathways was due to the development of genetic heterogeneity over time or due to selective pressure is unknown. Either way, it is possible the acquisition of RAS/RTK activating mutations could contribute to altered cellular responses to JAK2 inhibitors. The authors speculate that identifying such mutations in small cell populations within an individual patient could give insight into future trajectory of disease and provide a potential opportunity for altered clinical management to intervene in disease progression.

*Mass cytometry identification of MEK/ERK-dependent cytokine expression*—Using mass cytometry, Fisher et al. [85] detected and characterized expression of cytokines overproduced in myelofibrosis. Then, using ex vivo thrombopoietin (TPO) stimulation and treatment with ruxolitinib, the research team identified three subsets of cytokines as being differentially sensitive to TPO and ruxolitinib. First, a subset of cytokines, including CCL3/MIP-1α, CCL4/MIP-1β, and IL1RA, was identified to be induced by TPO, with ruxolitinib inhibiting this induction as well as basal levels of cytokine expression. A second group of cytokines, including TNF, IL-6, IL-8, and IL-10, was identified to be inducible by TPO, yet ruxolitinib did not inhibit basal levels of these cytokines. Finally, a third subset of cytokines, including TGFβ, VEGF, and IFNγ, was not induced by TPO and levels of these cytokines were insensitive to ruxolitinib. Given the role of inflammatory cytokines in myelofibrosis [42, 43] and the inefficacy of ruxolitinib to induce disease remission, the authors of this study further investigated the sensitivity of the expression of cytokines to small molecule inhibitors of pathways known to regulate cytokine expression. Subsequent analyses indicated that basal expression of many cytokines, including ruxolitinib insensitive cytokines, was inhibited by small molecule inhibitors of NFκB (pevonedistat) and MAP kinase (trametinib, JNKi8, and VX-745, inhibitors of MEK and JNK and p38 MAP kinases, respectively) activity. The study suggests that cytokine expression in myelofibrosis patients is differentially inhibited by ruxolitinib and that basal expression of many cytokines can be more effectively inhibited by targeting NFκB and MAPK activation. This provides additional mechanistic evidence that targeting the MEK/ERK pathway may enhance the efficacy of ruxolitinib by antagonizing inflammatory cytokine expression, as shown by Stivala et al. [65] who used MPN mouse models to show that combining MEK and JAK2 inhibition provides greater suppression of ERK target genes and cytokine expression. This concept is also supported by Brkic et al. [81] who showed genetic loss of ERK1/2 significantly enhanced suppression of cytokine expression in combination with ruxolitinib. Notably, the cytokines studied by Fisher et al. [85] were determined to be produced in monocytes, suggesting their contributions to disease phenotypes—and the effects of targeted therapies on cytokine expression—involve mechanisms that are extrinsic to disease driving stem cells. Given that cytokine suppression is inefficient with ruxolitinib alone, more direct inhibition of MEK/ERK signaling, as well as other pathways such as NFκB, may antagonize the pro-inflammatory and disease-driving state in MPNs [42, 43], and perhaps the ruxolitinib persistent survival of MPN-driving cells.

## Discussion

The consistent evidence, obtained using multiple experimental approaches, that activation of MEK/ERK contributes to the inefficacy/persistence of JAK2 inhibitor treatment in pre-clinical MPN studies is both interesting and disappointing from a therapeutic standpoint. Interesting because the consistent findings together strengthen the likelihood of its importance, and disappointing because while actionable there hasn’t been overwhelming success targeting this pathway to antagonize targeted therapy resistance. This is best exemplified by experience using MEK inhibitors in melanoma. Mechanisms of B-RAF inhibitor resistance commonly involve reactivation of RAF-mediated signaling, the immediate mediators of which are MEK/ERK activation [86]. As such, MEK inhibitors are approved for second line treatment of B-RAF inhibitor resistance in melanoma and the combination of B-RAF and MEK inhibition is currently the standard upfront targeted-therapy for *B-RAF*-mutated melanoma. However, positive effects of MEK inhibitor therapy in melanoma are transient, with disease progression generally seen within a year [87, 88]. Similarly, MEK inhibition is being assessed clinically to enhance the efficacy of EGFR inhibitor therapy in lung cancer, as both EGFR-intrinsic and extrinsic mechanisms of EGFR inhibitor resistance led to the activation of MEK/ERK signaling [89, 90]. One explanation of the persistent survival of lung cancer cells to EGFR inhibition is that while these inhibitors initially suppress MEK/ERK and AKT activity downstream of EGFR signaling, the suppression of AKT decreases the transactivation function of the ETS-1 transcription factor, leading to suppression of expression of dual-specificity phosphatases (DUSPs) [91–94] which negatively regulate ERK activity [95, 96]. Thus, loss of this negative regulation leads to paradoxical ERK activation due to an enhanced activation of ERK via signaling from non-EGFR pathways (e.g., via SRC) [91–93]. Whether or not dampened DUSP-mediated regulation of ERK activity plays a similar role in response to JAK2 inhibition is unknown, but could this contribute to how extrinsic growth factors maintain ERK activation during JAK2 inhibitor treatment [65]? Stivala et al. [65], in fact, demonstrate that DUSP4/6 mRNA is not suppressed in splenocytes or bone marrow cells of an MPN mouse model treated with ruxolitinib or MEK inhibition, suggesting that a loss of DUSP4/6 expression is not contributing to enhanced ERK activity in response to JAK2 or MEK inhibition in such therapeutic model systems. This dovetails nicely with ongoing work suggesting DUSP6 contributes to JAK2 inhibitor resistance and may be a therapeutic target in myelofibrosis [97], an unexpected finding given DUSP6 inhibition would in theory enhance ERK activity. Also, Stetka et al. [98] reported that cells expressing JAK2-V617F are addicted to DUSP1, which dephosphorylates JNK and p38 MAPK, in order to protect cells from inflammatory cytokines and DNA damage. The roles that DUSPs, whose canonical function is to antagonize MAPK activation [95, 96], may play in MPN remain to be fully understood, but these proteins have potential to be an exciting new avenue of research in MPN.

JAK2 inhibitor monotherapy is not a remission-inducing therapeutic approach, and as such, combination therapies may provide better upfront efficacy. Because JAK2 inhibition also has toxic effects (e.g., anemia, thrombocytopenia) that prevent many patients from obtaining the quality-of-life benefits from agents such as ruxolitinib, critical to such combination therapies will be the tolerability of the treatment regimen. This has been key to the promising results with BET and BCL-2 family inhibitor combinations with ruxolitinib in clinical trials. JAK2 inhibition combined with MEK or ERK inhibition, or perhaps inhibition of other mediators of ERK activation downstream of tyrosine kinase signaling such as SHP2 may antagonize the ERK-dependent cellular responses to ruxolitinib that overcome the apoptotic effects of ruxolitinib. It is noteworthy that while JAK2 and MEK inhibitor combination therapy in MPN mouse models significantly reduced bone marrow fibrosis it did not enhance a reduction in mutant allele burden [65], but it is possible that longer time of therapeutic treatment (a limitation of MPN mouse models) may show some effect in this regard. Excitingly, however, Jayavelu et al. [64] demonstrated that the combination of JAK2 and MEK inhibitor treatment induced reductions in JAK2-V617F allele burden in a xenograft model of primary MPN patient cells, and these results included evidence of molecular eradication of and selectivity for JAK2-V617F cells. This difference in the effect of JAK2 and MEK inhibitor combination therapy on mutant allele reduction could be due to the different therapeutic models employed—MPN mouse models driven by heterologous promoter overexpression of mutant MPN-driving proteins (e.g. MPL-W515L or JAK2-V617F) versus primary MPN patient cell xenografts. Primary MPN patient cell xenograft models, although being in an immune-deficient environment, may provide a superior option to MPN mouse models where disease is driven by heterologous, non-physiological, expression of MPN driver mutations, which may misrepresent disease driving signaling and cellular/disease dynamics (e.g. disease is polyclonal and driven by a single mutation), and response to experimental therapeutics. With that said it is worth noting that while MPN research has not readily been able to take advantage of NSG xenograft models for assessing primary MPN patient cell sensitivity to experiment therapeutics, advances in this space as demonstrated by Jayavelu et al. [64] and others [99] are providing exciting evidence that such assessment is possible and may afford distinct advantages over other commonly used MPN mouse models.

The phosphorylation/inactivation of BAD has been posited as a mediator of JAK2 inhibitor persistence downstream of both AKT and ERK activity [66], both effectors of JAK2 signaling [1, 100]. As phosphorylated BAD prevents it from negating the anti-apoptotic activity of BCL-2 family proteins [101, 102], the assessment of the BCL-2 protein inhibitor navitoclax in ongoing clinical studies [54] with ruxolitinib in myelofibrosis patients is relevant to the finding that BAD phosphorylation/inactivation plays a role in JAK2 inhibitor persistence [66, 83]. Notably, BCL-XL is the BCL-2 family member believed to be important in the JAK2 inhibitor persistent state [66, 83]. Data emanating from clinical studies indicate about 30% of patients given navitoclax, which inhibits BCL-XL and BCL-2, following failure of ruxolitinib treatment were able to achieve a 35% reduction in spleen volume with favorable impacts on MPN-driving allele burden and bone marrow fibrosis being observed as well [54]. As cellular response to ruxolitinib may lead to compensatory activation of ERK activity, combination of the more downstream inhibitors of MEK and BCL-2 proteins could augment the ability of JAK2 inhibitors to induce apoptosis in MPN-driving cells, with the caveat of the challenges of a triple combination therapy. This of course would be dependent on a therapeutic window whereby such cells display greater sensitivity to the combination treatment than their healthy counterparts and other cells.

In summary, numerous preclinical studies investigating mechanistic aspects of ruxolitinib persistence have highlighted a role of MEK/ERK activation in cell survival in the face of JAK2 inhibitor therapy [64–66]. Therapies that consist of inhibition of MEK/ERK in combination with JAK2 inhibition or perhaps with other promising targeted agents for MPN could improve JAK2 inhibitor therapy for MPN patients or lead to JAK2 inhibitor independent therapeutic approaches. The combination of JAK2 and ERK inhibition is being tested in a clinical trial (NCT04097821) for myelofibrosis [103] and perhaps combinations of MEK inhibition with BCL-2 inhibition, as well as combinations involving BET or PI3Kdelta inhibitors, could be effective therapeutic strategies in MPN. While MPN patients and their families continue to await the development of effective therapies, studies like those highlighted here should provide optimism that the ever-growing knowledge afforded by pre-clinical studies continues to illuminate exciting potential for clinical testing of novel therapeutic strategies to alter the natural course of MPN progression, ultimately leading to remission-inducing therapies.
